# Handlungsempfehlungen für die Gestaltung mobiler Apps in smarten und nachhaltigen Quartieren

**DOI:** 10.1365/s40702-021-00769-1

**Published:** 2021-08-17

**Authors:** Lukas Bonenberger, Valerie Graf-Drasch, Oliver Meindl

**Affiliations:** 1 Kernkompetenzzentrum Finanz- & Informationsmanagement, Hochschule Augsburg, Augsburg, Deutschland; 2 Lehrstuhl für Digitales Management, Universität Hohenheim, Stuttgart, Deutschland; 3 Kernkompetenzzentrum Finanz- & Informationsmanagement, Augsburg, Deutschland; 4 Projektgruppe Wirtschaftsinformatik, Fraunhofer FIT, Augsburg, Deutschland

**Keywords:** Smart City, Mobile Quartiers-Apps, Nachhaltigkeit, Benutzerzentrierte Softwareentwicklung, Energiekonsumverhalten, Smart City, Mobile District Apps, Sustainability, User-centric Software Engineering, Energy Consumption Behavior

## Abstract

Aktuell stammen zwei Drittel der weltweiten Energienachfrage sowie 70 % aller CO_2_-Emissionen aus Städten. Diese Tatsache bietet ein hohes Potenzial für das Adressieren des Klimawandels durch gezielte Maßnahmen in urbanen Gebieten. Der Bau oder die Sanierung energieeffizienter Gebäude, innovative Mobilitätskonzepte, nachhaltige Energieversorgung oder Anreizmechanismen für Bürger:innen helfen, Städte nachhaltiger und klimafreundlicher zu gestalten. Die dafür benötigten Services können digital durch sogenannte mobile Quartiers-Apps unterstützt und abgebildet werden. Mobile Quartiers-Apps helfen Bürger:innen ihr alltägliches Verhalten klimabewusster zu gestalten, können klassische Services digital abbilden und die Bürger:innen untereinander vernetzen. Bestehende mobile Quartiers-Apps sind meist individuell auf ein Stadtquartier zugeschnitten und adressieren ein Ziel aus den Bereichen Nachhaltigkeit, Services oder Soziales. Dieser Artikel stellt alle Zielbereiche von mobilen Quartiers-Apps auf eine generische Ebene und präsentiert neun Handlungsempfehlungen, die Städteplaner:innen bei der Konzeption und Entwicklung von mobilen Quartiers-Apps unterstützen sollen. Die Handlungsempfehlungen beleuchten die Dimensionen Benutzerzentriertheit, Datenschutz und Wirtschaftlichkeit. Alle diese Dimensionen greifen bei der Entwicklung von mobilen Quartiers-Apps ineinander und sollten für einen erfolgreichen Betrieb berücksichtigt werden. Der Artikel beschreibt die Umsetzung der Handlungsempfehlungen exemplarisch am Beispiel der Quartiers-App aus dem Verbundforschungsprojekt Stadtquartier 2050. In diesem Projekt sollen die Bewohner:innen zweier Demonstrationsquartiere in Deutschland klimaneutral mit Energie versorgt werden. Die App unterstützt dieses Projektziel durch die Bereitstellung von Werkzeugen zur Analyse und zum Benchmarking des Energiekonsumverhaltens der Benutzer:innen sowie die Information und Schulung der Bewohner:innen der Quartiere zu klimabewussterem Leben und Wohnen.

## Abstract (en)

Today, two-thirds of global energy demand and 70% of all carbon emissions originate from cities. These figures offer high potential for addressing climate change through targeted measures in urban areas. The construction or renovation of energy-efficient buildings, innovating mobility concepts, sustainable energy supply or concepts for incentivizing residents help to make cities more sustainable and climate-friendly. The services required in this regard can be supported and mapped digitally by so-called mobile district apps. Mobile district apps help residents of smart cities to shape their everyday behavior in a more climate-friendly and sustainable way, can digitally map classic services, and connect residents with each other. Existing mobile district apps are usually individually tailored to a smart city district and address a goal from one of the areas of sustainability, services, or community. This article brings all target areas of mobile district apps to a generic level and presents nine guidelines to support urban planners in the design and development of mobile district apps. The guidelines highlight the dimensions of user-centricity, data privacy, and economic feasibility. All these areas intertwine when developing mobile district apps and need to be considered for successful implementation. The article describes the realization of the guidelines using an app from the cooperative research project Stadtquartier 2050 concerning sustainable cities as an example. In this project, the residents of two demonstration districts in Germany are to be supplied with energy in a climate-neutral way. The app provides tools for analyzing and benchmarking users’ energy consumption behaviors, as well as educating residents on how to live more climate-consciously.

## Mobile Quartiers-Apps: Ein Sprungbrett für das Gelingen der Energie- und Wärmewende?

Heute lebt mehr als die Hälfte der globalen Bevölkerung in urbanen Gebieten (National Geographic 2021). Schätzungen zufolge soll sich dieser Anteil bis ins Jahr 2050 auf 70 % erhöhen (Brandt et al. 2018; United Nations 2018). Demnach wachsen Städte stetig. Jede Minute werden weltweit 10.000 m^2^ Stadtfläche gebaut (Weltwirtschaftsforum 2020). Alle fünf Tage entspricht dies etwa der Größe von Paris (Weltwirtschaftsforum 2020).

Das beschriebene urbane Wachstum bringt beachtliche Risiken aber auch Chancen mit sich: Einerseits sind bereits heute zwei Drittel der weltweiten Energienachfrage sowie 70 % der globalen CO_2_ Emissionen auf Städte zurückzuführen (Ribeiro et al. 2019; Weltwirtschaftsforum 2020; Gimpel et al. 2020a). Andererseits bilden Städte als Emissionsquelle gleichzeitig einen zentralen Ansatzpunkt, wenn es darum geht, den Klimawandel zu adressieren (Keller et al. 2019; Hosseini et al. 2018). Nicht ohne Grund ist das Streben nach *Smart and Sustainable Cities and Districts* eines der expliziten Ziele der sogenannten *Sustainable Development Goals* der Vereinten Nationen (United Nations 2015). Dementsprechend rückt das Thema Nachhaltigkeit zunehmend in den Fokus von Städteplaner:innen und städtischen Dienstleister:innen. Diese Ausrichtung ist jedoch hauptsächlich in ökonomischen Entscheidungen und Risikoabwägungen hinsichtlich der Folgen des Klimawandels begründet (Gouldson et al. 2015; Carbon Disclosure Project 2014; Hallegatte et al. 2013).

Auf städtischer Strategie-Ebene werden daher bereits verschiedene Konzepte verfolgt. Neben Energiekonzepten und technischen Maßnahmen beim Bau (z. B. Smart Home) oder bei der Modernisierung von Gebäuden spielen auch innovative Mobilitätskonzepte (z. B. E‑Mobilität) eine große Rolle (Hofmann et al. 2021). Darüber hinaus werden Konzepte zur Konsumverringerung in definierten Kontexten oder einer nachhaltigeren Energieversorgung (z. B. Mieterstrom) umgesetzt. All diese Ideen basieren jedoch auf der Annahme, dass die Bürger:innen die zur Verfügung gestellten Konzepte und Services in Anspruch nehmen. Dabei ist die Veränderung des individuellen Verhaltens ein zentraler Faktor, um klimabewusster zu leben und somit den Energieverbrauch zu reduzieren und Emissionen einzusparen (Hernández-López und González González 2019; Bitomsky et al. 2020; Henn und Kluge 2021). Eine individuelle Verhaltensänderung setzt einen Perspektivenwechsel von Städteplaner:innen und Dienstleistungsanbieter:innen voraus. So ist es essenziell, von der losgelösten Anbieter-Perspektive hin zu einer sektorübergreifenden, integrierten und bürgerzentrierten Nachfragesichtweise zu wechseln und dadurch die Bürger:innen in die Stadtentwicklung aktiv einzubinden (D’Onofrio et al. 2019; Hofmann et al. 2021; Pfäffli et al. 2018).

Aus diesem Grund werden auf Seiten der Städteplaner:innen zunehmend Projekte mit nachhaltigen und intelligenten Quartieren angegangen, welche die Bedürfnisse der Bewohner:innen besonders berücksichtigen. Ein nachhaltiges intelligentes Quartier umfasst einen Teilbereich einer Stadt, in dem unter anderem für die Bereiche Wirtschaft, Gesellschaft, Verwaltung, Mobilität, Umwelt, Energie und Wohnen zukunftsweisende Lösungen angewendet werden (Keller et al. 2019). Diese Lösungen bauen auf einer fortschrittlichen Informationstechnologie-Infrastruktur auf, die Vorteile für alle Akteure sicherstellt und dabei insbesondere eine hohe Lebensqualität für jede:n Bürger:in ermöglicht (Keller et al. 2019). Auf Seiten städtischer Dienstleister:innen entstehen zunehmend grüne und sektorübergreifende Informationssysteme, welche Verhaltensänderungen im Sinne der Nachhaltigkeit durch die Nutzung von smarten Dienstleistungen herbeiführen sollen (Gimpel et al. 2020b; Harnischmacher et al. 2020; Corbett und Mellouli 2017). Obwohl Stadtquartiere als auch grüne Informationssysteme einen wertvollen Beitrag zur Förderung eines klimabewussten Lebens und Wohnens innerhalb der Stadt leisten, fehlt derzeit ein gemeinschaftlicher Ansatz, welcher die beiden Kernideen – nachhaltige Quartiere und smarte Dienstleistungen – zusammenführt. Eine so entstehende holistische Perspektive könnte nicht nur Synergiepotenziale bei Städten und Dienstleister:innen heben, sondern insbesondere auch neue Services hervorbringen, welche nahe an den Bürger:innen entworfen wurden und so auf größere Akzeptanz stoßen das eigene Verhalten zu ändern.

Ein vielversprechender Ansatz zur Integration von smarten Services in einen nachhaltigen Stadt- beziehungsweise Quartierskontext sind mobile Quartiers-Apps (MQAs). MQAs sind Anwendungen auf mobilen Endgeräten der Bewohner:innen eines Stadtquartiers, welche das Leben und Wohnen der Bewohner:innen nicht nur vereinfachen, sondern auch im Sinne des Klimabewusstseins und der Nachhaltigkeit beeinflussen sollen (Brauer et al. 2016). Ursprung dieser MQAs sind Nachbarschafts-Apps, welche primär das Ziel verfolgen, eine reine Plattform zur Vernetzung innerhalb der Nachbarschaft zu bieten – eine Art digitales Schwarzes Brett mit Web 2.0-Funktionalitäten (Nextdoor 2021). Im Laufe der Zeit wurden auch Features zur Verwaltung der Infrastruktur innerhalb der Quartiere hinzugefügt, wie beispielsweise Reservierungen für Räume oder E‑Ladestationen (EUREF 2019). Aktuelle MQAs verfügen darüber hinaus über Funktionalitäten, welche in den Gebäuden verbaute Smart-Meter Technik nutzen, um beispielsweise eigene Energieverbräuche einzusehen oder die vernetzte Infrastruktur (z. B. Elektroladesäulen) innerhalb des Quartiers zu nutzen, um nachhaltige Shared-Services anzubieten (Animus 2021). Zusammenfassend ist das Ziel moderner MQAs, Bürger:innen besser mit und innerhalb des Quartiers zu vernetzen und zu einem klimabewussten Leben und Wohnen anzuregen beziehungsweise zu incentivieren. Bestehende MQAs sind daher meist sehr individuell auf die Bedürfnisse der Quartiere bzw. deren Bewohner:innen abgestimmt.

Die Entwicklung einer MQA stellt die Städteplaner:innen und städtischen Dienstleister:innen jedoch vor große Herausforderungen. Eine Konzeption von MQAs gliedert sich in drei relevante Dimensionen: *Benutzerzentriertheit, Datenschutz und Wirtschaftlichkeit.* Einerseits soll eine MQA Bewohner:innen anregen, sich klimabewusster zu verhalten und dabei eine möglichst attraktive Menge an Funktionalitäten besitzen, die von den Bewohner:innen genutzt werden. Andererseits soll die Entwicklung den Prinzipien der Wirtschaftlichkeit folgen und einen konkreten Nutzen für alle beteiligten Interessensgruppen stiften. Gleichzeitig soll dabei geltendes Recht eingehalten werden, welches insbesondere bei der Verarbeitung von Daten aus dem Smart Home respektive den einhergehenden Smart-Metern zu beachten ist.

Daher stellt sich für Städteplaner:innen und städtische Dienstleister:innen nicht nur die Frage, welche Ziele eine MQA im Sinne des Klimabewusstseins und der Nachhaltigkeit verfolgen könnte. Es ist ebenso von zentraler Bedeutung, wie die drei genannten Dimensionen bei der Entwicklung einer MQA angegangen oder gelöst werden können. Genau diese Fragestellungen werden im öffentlich geförderten Projekt Stadtquartier 2050 adressiert und im Zuge einer MQA in zwei Demonstrationsquartieren beispielhaft erprobt. Die aus dem Projekt abgeleiteten übertragbaren Handlungsempfehlungen sollen künftigen Quartiersprojekten dazu dienen, MQAs mit Fokus auf die Benutzer:innen zu entwickeln, datenschutzkonform auszurollen und unter ökonomischen Gesichtspunkten zu designen.

## Modernes und klimabewusstes Leben und Wohnen: Das Projekt Stadtquartier 2050

Ziel des Projekts Stadtquartier 2050 ist es, die Bewohner:innen zweier Demonstrationsquartiere in Stuttgart und Überlingen klimaneutral mit Energie zu versorgen. Um dieses Ziel sozial verträglich umzusetzen, werden die Bewohner:innen bereits beim Bau beziehungweise der Sanierung der insgesamt 960 Wohneinheiten mit einer Gesamtinvestitionssumme von circa 190 Mio. € in das Vorhaben mit einbezogen. Informationsveranstaltungen dienen dazu, bereits vor dem Vorhaben den Dialog zu suchen und auf die Bedürfnisse und Fragen der Bewohner:innen einzugehen. Daneben sollen die Bewohner:innen jedoch auch die Möglichkeit haben, aktiv zum Ziel der Klimaneutralität beitragen zu können. Hierzu wird eine innovative MQA entwickelt, die darauf abzielt, ein klimabewusstes Leben und Wohnen der Bewohner:innen zu unterstützen und somit eine klimaneutrale Energieversorgung zu katalysieren. Die MQA wird dabei inkrementell und iterativ entwickelt, um mit relevanten Interessensgruppen innerhalb des Projekts in engem Austausch zu bleiben.

**Abb. 1:**
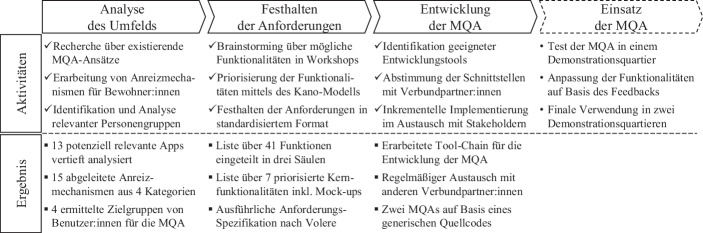
Entwicklungsprozess der MQA im Projekt Stadtquartier 2050

Der Entwicklungsprozess selbst lässt sich in mehrere Phasen unterteilen (siehe Abb. 1), welche speziell darauf abgestimmt sind, eine bewohnerzentrierte Entwicklung zu fördern:

- Die Phase *Analyse des Umfelds* trägt dazu bei, technische und soziologische Vorarbeiten zu leisten. Bestehende App-Ansätze werden analysiert, Zielgruppen von Benutzer:innen literaturgestützt aufbereitet und projektspezifische Anreizmechanismen entwickelt.
- In der Phase *Festhalten der Anforderungen* wird Wissen von Expert:innen innerhalb mehrerer Workshops aufgearbeitet, sodass eine Priorisierung (Kano 1984) und Spezifikation (Robertson und Robertson 2012) geeigneter App-Funktionalitäten möglich sind.
- Die *Entwicklung der App* dient dazu, die Erkenntnisse aus den vorangegangenen Phasen sowie die Ergebnisse beteiligter Projektpartner in die Entwicklung einer MQA einfließen zu lassen. Hierbei wird ein intensiver Austausch angestrebt, um interdisziplinäres Wissen innerhalb des Projekts Stadtquartier 2050 zu teilen.
- Beim *Einsatz der App *wird die entwickelte MQA in den zwei Demonstrationsquartieren in Stuttgart und Überlingen ausgerollt und betrieben.

Im Zuge der Gestaltung und Entwicklung der MQA wurden neun Handlungsempfehlungen abgeleitet, welche auf andere Quartiersprojekte übertragbar sind. Sie werden im folgenden Kapitel vorgestellt.

## Konzeption und Entwicklung erfolgreicher mobiler Quartiers-Apps: Handlungsempfehlungen

Nicht nur im vorangegangenen Kapitel vorgestellten spezifischen Projekt Stadtquartier 2050, sondern allgemein in aktuellen Quartiersprojekten wie beispielsweise EnStadt:Pfaff (Pfaff Reallabor 2021) werden mobile Apps entwickelt und betrieben, um dem Klimawandel durch technisch unterstütztes klimabewusstes Verhalten entgegenwirken zu können. Die möglichen Ziele für den Einsatz einer MQA sind vielfältig. Sie reichen von der Analyse und dem Benchmarking des Energiekonsumverhaltens der Bewohner:innen oder deren Information und Schulung zu klimabewussterem Leben über die Unterstützung innovativer Mobilitätskonzepte für das Quartier bis hin zur Bereitstellung digitaler Verwaltungsservices für die Bewohner:innen oder eine Förderung der Interaktion zwischen Bewohner:innen und damit des sozialen Lebens im Quartier, wie zum Beispiel bei der App Animus (Animus 2021).

Aus den Erfahrungen im Projekt Stadtquartier 2050 wurden die drei Dimensionen Benutzerzentriertheit, Datenschutz und Wirtschaftlichkeit identifiziert, die für die Entwicklung einer MQA und die daraus abgeleiteten Handlungsempfehlungen zentral sind. Unterstützung für die Bedeutung der Dimensionen findet sich auch in der Literatur. Sepasgozar et al. (2019) beschreiben die Wichtigkeit der Benutzerzentriertheit zur Erreichung von Technologieakzeptanz im urbanen Kontext, Vandercruysse et al. (2020) betonen die Bedeutung von Datenschutz für Services in smarten Städten und Bastidas et al. (2018) und Weiß und Strahringer (2021) greifen das Thema Wirtschaftlichkeit bei der Entwicklung von digitalen Artefakten und Services im städtischen Umfeld auf.

**Abb. 2:**
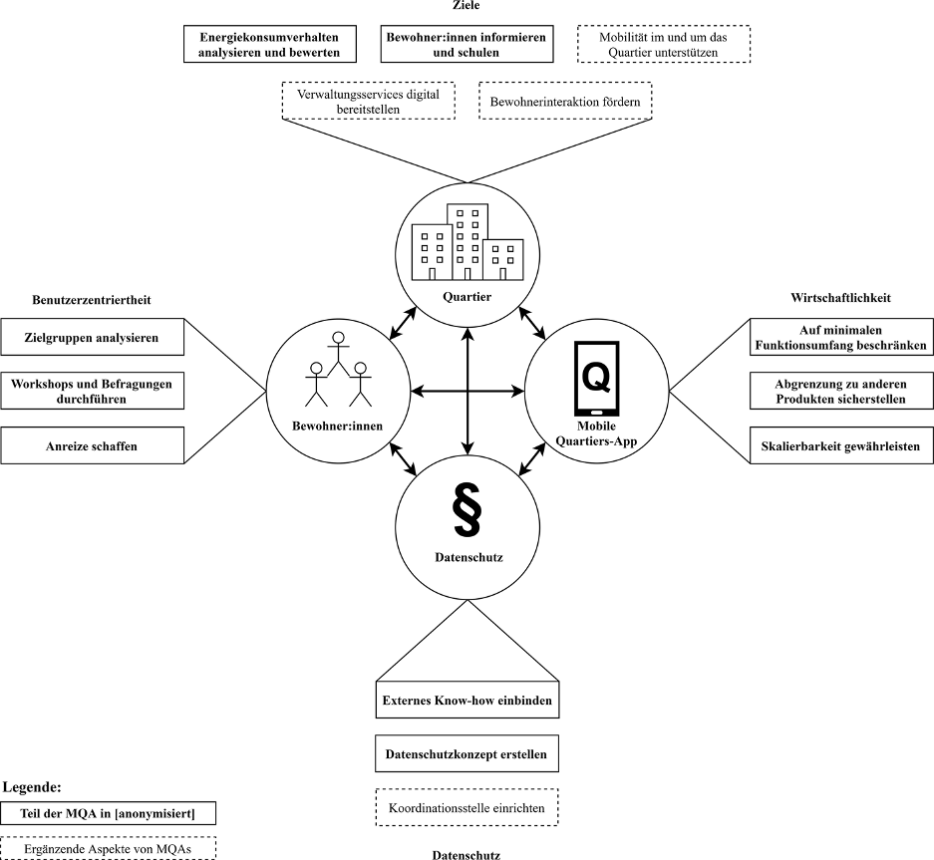
Zusammenspiel zwischen Bewohner:innen, einer MQA, dem Quartier sowie der Dimensionen Benutzerzentriertheit, Datenschutz und Wirtschaftlichkeit

Alle der oben genannten Dimensionen dienen einer Verbesserung der Situation der Bewohner:innen eines Quartiers, welche zugleich die Benutzer:innen einer MQA sind. Die erste Dimension der Handlungsempfehlungen für die Konzeption und Entwicklung erfolgreicher MQAs in diesem Beitrag ist daher ein benutzerzentriertes Vorgehen. Abb. 2 fasst mögliche Ziele einer MQA sowie das Zusammenspiel zwischen Bewohner:innen, der MQA, dem Quartier sowie den drei Dimensionen der Handlungsempfehlungen grafisch zusammen. Die fett gedruckten Aspekte finden sich in der MQA aus dem Projekt Stadtquartier 2050 wieder. Diese wird als Fallbeispiel in Kapitel 4 näher vorgestellt.

### Benutzerzentriertheit

Alle vorgestellten Ziele von MQAs sind nur erreichbar, wenn die Bewohner:innen die zur Verfügung gestellten Konzepte und Services auch in Anspruch nehmen. Nachhaltigkeitsziele können insbesondere durch die Veränderung des individuellen Verhaltens erreicht werden. Konzeption und Entwicklung einer MQA sollten daher benutzerzentriert erfolgen, um durch eine hohe Benutzerakzeptanz Verhaltensänderungen herbeiführen zu können. Für die benutzerzentrierte Softwareentwicklung ist eine Analyse relevanter Personengruppen erforderlich. Neben einer Ausrichtung der Ziele und Anforderungen einer MQA auf eine oder mehrere dieser Zielgruppen ist eine direkte Einbindung der Benutzer:innen in das Anforderungsmanagement sinnvoll. Diese kann beispielsweise durch Befragungen mithilfe von Fragebögen oder auch im Dialog erfolgen, beispielsweise durch Fokusgruppen, also moderierte Gruppendiskussionen mit den Quartiersbewohner:innen. Evaluationen durch Benutzer:innen sollten zu mehreren Zeitpunkten stattfinden. Zum einen vor der Entwicklung, beispielsweise in initialen Anforderungsworkshops, zum anderen nach der Anforderungserhebung auf Basis bereits entwickelter Konzepte. Durch die Implementierung von Anreizmechanismen aus den vier definierten Kategorien *Bequemlichkeit/Salienz, Informationsvermittlung, Monitoring/Feedback* und *sozialer Einfluss* (Abrahamse und Schuitema 2020; Heiskanen et al. 2020) kann die Benutzerzentriertheit einer MQA weiter gestärkt werden.

### Datenschutz

Datenschutz ist die zweite zentrale Dimension der Handlungsempfehlungen für Konzeption und Entwicklung erfolgreicher MQAs. Bei der Kommunikation mit Bewohner:innen smarter Stadtquartiere werden Kontaktdaten erfasst. Möglicherweise werden Energieverbräuche oder Raumklimadaten der Bewohner:innen auf Haushaltsebene erfasst. Für die Bereitstellung bestimmter Services im Quartier oder Forschungs- und Entwicklungsaktivitäten wie beispielsweise Befragungen oder Workshops kann die Erfassung soziodemografischer Daten erforderlich sein. Mit diesen Daten könnten Profile gebildet werden, die Rückschlüsse auf die Gewohnheiten und Lebensumstände der Bewohner:innen zulassen. Viele Unternehmen nutzen die Unachtsamkeit von Konsument:innen aus, die zwar angeben, auf Datenschutz Wert zu legen, jedoch keine Maßnahmen dazu ergreifen. Dabei handelt es sich um das sogenannte Privatsphäre-Paradoxon (Lasarov und Hoffmann 2021). Nicht nur aufgrund des gesetzlichen Einhaltungszwangs, sondern auch zur Sicherstellung der Akzeptanz der Benutzer:innen sollten daher über die gesamte Laufzeit des Entwicklungsprojekts beziehungsweise die Lebensdauer einer MQA Datenschutzmaßnahmen ergriffen werden. Bei Vernachlässigung drohen neben hohen Bußgeldern auch das Scheitern des Projekts oder Teilen davon durch fehlende Akzeptanz der Bewohner:innen.

Um im Bereich Datenschutz möglichst sorgfältig zu arbeiten und eine hohe Qualität der Ergebnisse sicherzustellen, empfiehlt sich die Einbindung externen Know-hows im Bereich Datenschutzrecht, wenn im Entwicklungsteam einer MQA beziehungsweise im Forschungsprojekt keine Expert:innen dieser Domäne vertreten sind. Vor allem bei der Erstellung und kritischen Prüfung von Datenschutzdokumenten wie Datenschutzkonzept, Informationsschriften oder Einwilligungserklärungen im Hinblick auf Konsistenz sowie einer rechtlich sicheren Implementierung von Prozessen rund um eine MQA kann die Einbindung externen Know-hows den langfristigen Projekterfolg sichern.

Bei jeder Verarbeitung personenbezogener Daten bewegt man sich nach EU-Datenschutzgrundverordnung (DSGVO) grundsätzlich im Ausnahmebereich (Europäische Union 2019). Für jede Verarbeitung muss daher mindestens eine der in Art. 6 DSGVO genannten Rechtsgrundlagen vorliegen, wie beispielsweise die Einwilligung in die Verarbeitung oder eine Wahrung berechtigter Interessen des Verantwortlichen. Unabhängig von der Wahl der Rechtsgrundlage für die Datenverarbeitung ist die Erstellung eines Datenschutzkonzepts ratsam. Dieses beschreibt neben den Entscheidungen zur Einhaltung der einschlägigen Rechtsvorschriften auch technisch-organisatorische Schutzmaßnahmen zur Aufrechterhaltung des Datenschutzes und der Informationssicherheit bei der Entwicklung einer MQA.

Um den Datenschutz von Projektbeginn an zentral zu managen und Vorschläge und Entscheidungen rechtzeitig und regelmäßig an alle Interessensgruppen des Projekts zu kommunizieren, ist die Einrichtung einer zentralen Datenschutzkoordinationsstelle für den laufenden Projektbetrieb zu Beginn des Projekts ratsam. Aufgabe der Datenschutzkoordinationsstelle ist die zentrale Kommunikation von Anliegen der Bewohner:innen, beispielsweise bei Geltendmachung der Betroffenenrechte nach Art. 15–18 DSGVO. Selbst wenn sich Bewohner:innen bei gemeinsam Verantwortlichen zum Datenschutz nach Art. 26 DSGVO mit einem Auskunftsersuchen an einen beliebigen Projektpartner wenden, kann dieser das Anliegen unaufwändig an die Datenschutzkoordinationsstelle kommunizieren, welche es wiederum an die verantwortlichen Partner weiterleitet.

### Wirtschaftlichkeit

Um MQAs auch außerhalb von Konsortial- oder Forschungsprojekten entwickeln und betreiben zu können, sollten einige wirtschaftliche Aspekte berücksichtigt werden. Einer der zu berücksichtigenden Aspekte ist der Funktionsumfang einer MQA. Der Funktionsumfang sollte auf Grundlage der festgelegten Ziele der MQA definiert werden. Die Bereitstellung innovativer und nachhaltigkeitsunterstützender Funktionalitäten soll beispielsweise das Quartier aufwerten und dadurch die Attraktivität für Kauf oder Miete von Immobilien im Quartier erhöhen. Wenn dies in einem Demonstrationsumfeld, beispielsweise im Rahmen eines Forschungsprojekts, gelingt, ist eine vergütete Überlassung der Nutzungsrechte einer MQA von einem öffentlich geförderten Geldgeber zu einem privaten Quartiersbetreiber denkbar. In diesem Fall profitieren beide Parteien von der Entwicklung der App. Eine allgemeine Funktionalität zur Sicherstellung der Rentabilität einer MQA ist das Einbinden lokaler Zielgruppenwerbung. Beispielsweise könnten Läden oder Handwerksbetriebe im oder nahe des Quartiers potenzielle Kund:innen so direkt auf sich aufmerksam machen.

Neben der Definition eines sinnvollen Funktionsumfangs sollten dringend mögliche Redundanzen zu bereits existierenden Lösungen wie anderen Apps oder Online-Diensten vermieden werden. Solche Redundanzen können ein K.-o.-Kriterium für die Profitabilität einer MQA sein. Funktionalitäten zur sozialen Verbindung von Bewohner:innen untereinander können beispielsweise oftmals durch bereits bestehende Softwarelösungen wie soziale Netzwerke deutlich kostengünstiger umgesetzt werden. Eine Integration solcher Features kann zwar eine Erleichterung für die Bewohner:innen darstellen, die jedoch den Mehraufwand bei der App-Entwicklung nicht aufwiegt. Insgesamt sollten in einer MQA also nur Features realisiert werden, die nicht über Standardsoftware abgedeckt werden können. Im Kontext von smarten Quartieren sind dies vor allem die Integration und Visualisierung von Smart-Meter-Daten, da es hierzu aufgrund der geringen Verbreitung von MQAs und der hohen Individualität und Heterogenität der Messinfrastrukturen in einzelnen Quartieren keine geeignete Standardsoftware gibt.

Ein weiterer Aspekt in Punkto Wirtschaftlichkeit ist eine hohe Skalierbarkeit. Da MQAs als Softwareprodukte grundsätzlich sehr niedrige Grenzkosten haben, können die hohen Entwicklungskosten vor allem durch Mehrfachanwendung kompensiert werden. Im besten Fall wird eine MQA in vielen verschiedenen Quartieren eingesetzt. Um solche Mehrfachanwendungen zu ermöglichen, sollte das im letzten Absatz beschriebene Problem der Individualität und Heterogenität der Informationstechnologie-Infrastrukturen in den Quartieren bewältigt werden. Dies kann über die generische Definition und Realisierung von Funktionalitäten und Schnittstellen erfolgen. Das betrifft vor allem die Schnittstellen zur Sensortechnik in den Quartieren, falls die MQA zur Energieverbrauchsanalyse eingesetzt wird. Auch die Ziele der MQA sollten für einen skalierbaren Einsatz der MQA quartiersunabhängig sein. Insgesamt soll ein Kompromiss zwischen einer möglichst hohen Erfüllung der Bedürfnisse der Bewohner:innen und einer möglichst hohen Rentabilität bei Entwicklung und Betrieb einer MQA geschaffen werden.

### Zusammenspiel als Leitfaden

Die vorgestellten, aus Projekterfahrungen abgeleiteten Handlungsempfehlungen aus den Dimensionen Benutzerzentriertheit, Datenschutz und Wirtschaftlichkeit bilden zusammen einen Leitfaden zur Entwicklung erfolgreicher MQAs. Jedoch lassen sie auch im Zusammenspiel viel Spielraum für die App-Entwicklung, nicht jeder Einzelfall ist abgedeckt. Für bestimmte Anwendungsfälle soll der Leitfaden jeweils um fallspezifische Aspekte ergänzt werden. Durch das Befolgen der Handlungsempfehlungen bei der Entwicklung von MQAs kann der Erfolg und dadurch die Benutzerzahl der App erhöht werden. Hieraus resultiert bei Apps mit Klima- und Nachhaltigkeitszielen ein positiver transitiver Effekt für das Gelingen der Energie- und Wärmewende. Tab. 1 fasst die vorgestellten Handlungsempfehlungen auf einen Blick zusammen.

**Tab. 1:** Handlungsempfehlungen für Konzeption und Entwicklung erfolgreicher MQAs in den Dimensionen Benutzerzentriertheit, Datenschutz und Wirtschaftlichkeit

*Benutzerzentriertheit*	
**H1** Gruppen von Benutzer:innen	Hohe Akzeptanz ist ein essenzielles Erfolgskriterium für MQAs. Um die Akzeptanz der Bewohner:innen sicherzustellen, ist eine Analyse relevanter Personengruppen erforderlich
**H2** Einbindung von Benutzer:innen	Die Benutzer:innen der MQA sollten in das Anforderungsmanagement bei der Entwicklung eingebunden werden. Dies kann beispielsweise durch Befragungen oder Fokusgruppen erfolgen
**H3** Anreizmechanismen	Die MQA soll Anreizmechanismen für die Benutzer:innen enthalten. Durch diese wird die Benutzerzentriertheit der App weiter gestärkt
*Datenschutz*	
**H4** Einbindung externen Know-hows	Die Einbindung externen Know-hows im Bereich Datenschutzrecht kann durch Überprüfung und Qualitätssicherung von Datenschutzdokumenten und -prozessen den Projekterfolg sichern
**H5** Datenschutzkonzept	Basierend auf der jeweiligen Rechtsgrundlage für die Datenverarbeitung soll ein passendes Datenschutzkonzept für die MQA entwickelt und umgesetzt werden
**H6** Koordinationsstelle	Die Einrichtung einer zentralen Koordinationsstelle für den Datenschutz bei Entwicklung und Betrieb der MQA ist sinnvoll
*Wirtschaftlichkeit*	
**H7** Funktionsumfang	Der Funktionsumfang der MQA sollte sich an den Zielen der App orientieren und auf ein wirtschaftlich sinnvolles Maß begrenzt bleiben
**H8** Redundanzfreiheit	Redundanzen zu bereits existierenden Apps oder Diensten sollten vermieden werden, da sie die Wirtschaftlichkeit und damit den Projekterfolg gefährden
**H9** Skalierbarkeit	Schnittstellen sollten generisch ausgestaltet werden, um eine Skalierbarkeit der MQA zu gewährleisten. Diese Eigenschaft ist für einen wirtschaftlichen Betrieb erforderlich

## Umsetzung der Handlungsempfehlungen am Fallbeispiel der Quartiers-App im Projekt Stadtquartier 2050

Die in Kapitel 3 vorgestellten Handlungsempfehlungen beschreiben ein generisches Vorgehen für die Konzeption und Entwicklung erfolgreicher MQAs. Dieses Kapitel veranschaulicht, wie die Umsetzung der Handlungsempfehlungen in der MQA im Projekt Stadtquartier 2050 zum Projektziel des klimaneutralen Lebens und Wohnens in den Demonstrationsquartieren beiträgt.

Aus den in Abb. 2 dargestellten möglichen Zielen einer MQA fokussiert die App im Projekt Stadtquartier 2050 die Ziele *Analyse und Benchmarking von Energiekonsumverhalten* sowie die *Information und Schulung der Bewohner:innen *zu einem klimabewussteren Leben und Wohnen. Beide Ziele sollen die Bewohner:innen der Demonstrationsquartiere im Projekt dazu bewegen, ihr Verhalten klimafreundlicher zu gestalten. Da diese Ziele stark benutzerbezogen sind, wurde im Projekt eine Analyse relevanter Zielgruppen von Benutzer:innen durchgeführt (H1). Diese basieren auf den Energiekulturen von Barton (2013, S. 23) und repräsentieren Gruppen von Energieverbrauchern in einem westlichen Milieu. Mögliche Zielgruppen, die für eine MQA infrage kommen, sind auf Grundlage dieser Energiekulturen:

- *Singles oder Paare unter 40 Jahren* mit geringer Kaufkraft, mittlerer Bildung und Umweltbewusstsein, die sich mit dem Klimawandel auskennen und erfolgreich Energiesparmaßnahmen anwenden.
- *Singles oder Paare im Alter von 30 bis 60 Jahren* mit mittlerer Kaufkraft und Bildung, Sparsamkeit als Wertvorstellung, die viele Energiesparmaßnahmen beachten und sich gerne über Energiethemen austauschen.
- *Paare oder Eltern im Alter von 30 bis 50 Jahren* mit hoher Kaufkraft und mittlerem Bildungsgrad, Glück und Freude im Leben als Wertvorstellung, hohem Energieverbrauch und niedrigem monetärem Anreiz, Energie zu sparen.
- *Personen über 50 Jahren* mit mittlerer bis hoher Kaufkraft und mittlerer Bildung, Bequemlichkeit als Wertvorstellung, wenig Sorgen vor dem Klimawandel, niedriger Technikaffinität und niedriger Motivation zum Energiesparen.

Die MQA im Projekt Stadtquartier 2050 zielt auf die ersten drei dieser Benutzergruppen ab. Personen aus der vierten Zielgruppe (Personen über 50) sind keine Hauptzielgruppe der App. Da sie tendenziell weder technische Affinität noch einen relevanten Energiesparwillen aufweisen, werden sie bei der Umsetzung der Ziele der App nicht besonders berücksichtigt.

Die zentralen Anforderungen der Bewohner:innen an die App wurden früh im Projekt in zwei Anforderungsworkshops (Rupp und die SOPHISTen 2014, S. 113) mit den Projektpartnern ermittelt. Sie gliederten sich in die Bereiche Services, Soziales und Verwaltung und umfassen Themen wie Energiesparen, Energieverbräuche, Mobilität, Verbräuche im Quartier, Interaktion zwischen Bewohner:innen, digitale Wohnungsakten und Möglichkeiten für Reparatur-/oder Störungsmeldungen. Die Perspektive der Bewohner:innen wurde in diesen Workshops vor allem durch die Baugenossenschaft eines der Demonstrationsquartiere vertreten. Zusätzlich erfolgt die Einbindung der Bewohner:innen in den Entwicklungsprozess der MQA im Projekt Stadtquartier 2050 parallel zur App-Entwicklung durch Fokusgruppen (H2) um die initial ermittelten Anforderungen zu prüfen und gegebenenfalls anzupassen. Ein Vorschlag, der in diesem Rahmen bereits geäußert wurde, ist die Einbindung bestehender lokaler Infrastruktur, beispielsweise des Quartierscafés, in die App.

Im Projektverlauf wurden zudem Anreizmechanismen zum Energiesparen für die Bewohner:innen ermittelt, die über die App transportiert werden können (H3). Diese sind:

- *Energy Reports*, die den Bewohner:innen Details zu ihrem Energieverbrauch aufzeigen
- *Cost Savings Reports*, die den Bewohner:innen aufzeigen, wie viel Geld sie bei einer Anpassung ihres Energiekonsumverhaltens einsparen könnten
- *Gamification-Elemente*, welche einen produktiven Wettbewerb um einen möglichst niedrigen Energieverbrauch zwischen den Bewohner:innen fördern
- Allgemeine *Tipps & Tricks*, um im Alltag klimabewusst und umweltschonender zu leben

Dem Datenschutz wird im Projekt Stadtquartier 2050 eine hohe Bedeutung zugemessen. Die MQA im Projekt verarbeitet neben Energieverbrauchsdaten auf Haushaltsebene auch Wohnklimadaten, Nutzungsdaten und soziodemografische Daten der Benutzer:innen. Bei all diesen Daten handelt es sich um schützenswerte personenbezogene Daten. Neben einem Lieferobjekt zur Aufbereitung des Datenschutzes beim Umgang mit feingranularen Strom- und Wärmeverbrauchsdaten gibt es im Projekt Stadtquartier 2050 ein Lieferobjekt zur Aufbereitung des Datenschutzes im Rahmen von MQAs. Dieses Lieferobjekt beschäftigt sich explizit mit dem Datenschutz bei der Entwicklung und dem Betrieb einer MQA (H4). Im Fokus dieses Lieferobjekts steht die Erstellung und Umsetzung eines umfangreichen Datenschutzkonzepts, welches die Bedürfnisse der Bewohner:innen durch die Einholung der Einwilligung zur Verarbeitung gemäß Art. 6 Abs. 1 UAbs. 1 lit. a DSGVO und die Einrichtung der für den Datenschutz gemeinsam Verantwortlichen Projektpartner gemäß Art. 26 DSGVO durch eine Datenschutzvereinbarung berücksichtigt (H5). Durch das Befolgen der Handlungsempfehlungen H4 und H5 mit hoher Transparenz und maximaler Freiwilligkeit für die Bewohner:innen sollten Datenschutzbedenken proaktiv ausgeräumt werden. Da bei der Verarbeitung personenbezogener in der MQA des Projekts Stadtquartier 2050 höchste Datenschutzanforderungen und Freiwilligkeit gelten, wurde die Sensibilität der Bewohner:innen auf dieses Thema nicht gesondert untersucht. Erfahrungen aus dem Projekt zeigen zudem, dass die Einrichtung einer koordinierenden, projektinternen Stelle für den Datenschutz für projektübergreifende Datenschutztätigkeiten und die zentrale Bearbeitung von Anfragen von Projektbeginn an ratsam ist, um Missverständnisse auszuräumen, den Koordinationsaufwand zu reduzieren und den Projektfortschritt sicherzustellen (H6).

Die ermittelten Anforderungen wurden auf Basis des Kano-Modells (Kano 1984) priorisiert und die wichtigsten als Kernfunktionalitäten für die Entwicklung der MQA berücksichtigt (H7). Durch die iterative Ausarbeitung der Funktionalitäten in Kombination mit der Recherche über bereits existierende Ansätze konnten Redundanzen in der MQA zu bereits verfügbaren Systemen vermieden werden (H8). Auch die Ausgestaltung generischer Schnittstellen ist für die App bereits durch das Projekt Stadtquartier 2050 vorgegeben. Da es im Projekt zwei voneinander unabhängige Demonstrationsquartiere gibt, wird für die Anbindung der übergreifenden MQA eine generische Schnittstelle spezifiziert und entwickelt, die eine gleichwerte Anbindung der Messdatenserver beider Demonstrationsquartiere ermöglicht (H9). Durch die Umsetzung diese Handlungsempfehlungen wird die wirtschaftliche Attraktivität für den Weiterbetrieb der App nach der Laufzeit des Verbundvorhabens angestrebt. Für diesen Weiterbetrieb gibt es bereits konkrete Interessenten.

**Abb. 3:**
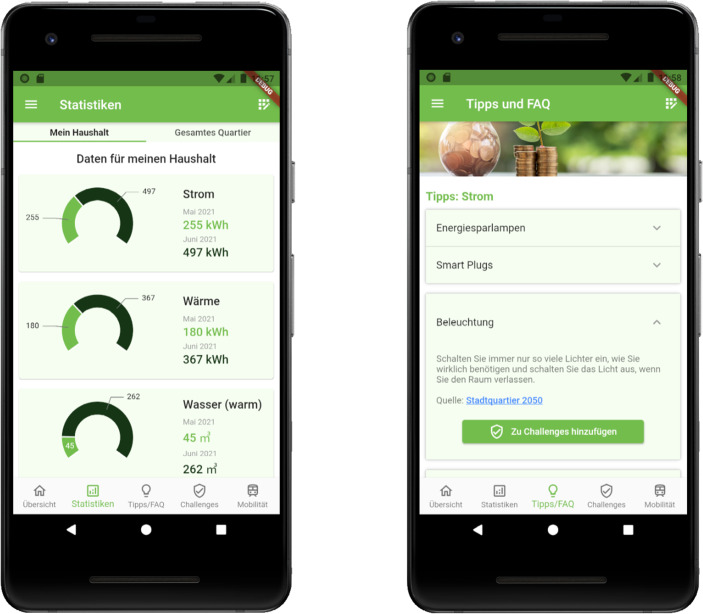
Screenshots der MQA im Projekt Stadtquartier 2050. **a** Visualisierte Stromverbrauchsdaten (randomisiert generierte Beispieldaten); **b** Energietipps

Abb. 3 zeigt beispielhaft zwei Screenshots aus der MQA im Projekt Stadtquartier 2050 im Entwicklungsstadium. Links ist das Feature Energieverbrauchsanalyse zu sehen, welches das Ziel Analyse des Energiekonsumverhaltens umsetzt. Rechts ist das Feature Tipps abgebildet, welches das Ziel der Information und Schulung der Bewohner:innen zu klimabewussterem Leben und Wohnen adressiert. Tab. 2 stellt abschließend die Maßnahmen bei der Entwicklung der MQA den jeweiligen allgemeinen Handlungsempfehlungen für Konzeption und Entwicklung erfolgreicher MQAs aus Kapitel 3 gegenüber.

**Tab. 2:** Maßnahmen zur Umsetzung der Handlungsempfehlungen in der MQA im Projekt Stadtquartier 2050

*Handlungsempfehlung*	*Maßnahmen*
**H1** Gruppen von Benutzer:innen	Analyse von Zielgruppen für die MQA und Ausrichtung der App auf drei Zielgruppen
**H2** Einbindung von Benutzer:innen	Durchführung von Anforderungsworkshops mit Vertreter:innen von Interessen der Bewohner:innen und Fokusgruppen mit den Bewohner:innen zur Gewinnung von Feedback für Funktionsanpassungen
**H3** Anreizmechanismen	Implementierung der Anreizmechanismen Home Energy Report, Cost Savings Report, Energiespartipps und Gamification-Elemente in der MQA
**H4** Einbindung externen Know-hows	Einbindung von Datenschutzexpert:innen für Beratungen zu adäquaten kontextbezogenen Datenschutzmaßnahmen und Qualitätssicherung projektbezogener Datenschutzdokumente
**H5** Datenschutzkonzept	Erstellung und Umsetzung eines umfangreichen Datenschutzkonzepts inklusive einer Vereinbarung zum Datenschutz zwischen den Projektpartnern
**H6** Koordinationsstelle	Bündelung der Lieferobjekte zum Datenschutz bei einem Projektpartner
**H7** Funktionsumfang	Durchführung von Workshops zur Anforderungsermittlung und Anwendung bewährter Priorisierungsverfahren
**H8** Redundanzfreiheit	Redundanzvermeidung durch Recherche über bestehende Lösungen und iterative Entwicklung der MQA
**H9** Skalierbarkeit	Entwicklung generischer Schnittstellen, um die MQA in unabhängigen Quartieren einsetzen zu können

Die überwiegende Zahl der in diesem Artikel vorgestellten Handlungsempfehlungen wurde bei der Entwicklung der MQA im Projekt Stadtquartier 2050 über die gesamte Projektlaufzeit wie beschrieben erfolgreich umgesetzt. Da die Entwicklung der App hauptsächlich während der COVID-19-Pandemie erfolgte, konnte H2 jedoch nicht so intensiv wie initial geplant umgesetzt werden. Die teils sehr strikten Kontaktbeschränkungen erschwerten den Kontakt zu den Bewohner:innen für eine starke Einbindung und Interaktion. Die reduzierten Kontaktmöglichkeiten zu den Bewohner:innen wurden jedoch durch Wissen von Expert:innen ausgeglichen. In Bezug auf H6 erfolgte erst während der der Laufzeit des Projekts der Erkenntnisgewinn, dass die Einrichtung einer Koordinationsstelle für den Datenschutz bereits zu Projektbeginn ratsam ist. Dennoch war zumindest die Erstellung aller Lieferobjekte für den Datenschutz der MQA bei einem Projektpartner gebündelt.

## Mobile Quartiers-Apps: Ein möglicher Lösungsansatz

Mit wachsender Größe und Bedeutung sollten Städte sich noch aktiver im Sinne der Nachhaltigkeit einsetzen, um die Folgen des Klimawandels einzugrenzen. So liegt es langfristig an den Gestalter:innen der Smart Cities von morgen, nicht nur top-down getriebenen Projekte zu fokussieren, welche aus ökonomischen Entscheidungen und Risikoabwägungen hinsichtlich der Folgen des Klimawandels zum Selbstschutz durchgeführt werden. Es bedarf auch bottom-up-Initiativen, die sich bei der Gestaltung proaktiv mit den Bewohner:innen auseinandersetzen, um neue, ganzheitlich gedachte und sektorübergreifende Lösungen für Quartiere zu entwickeln. Eine wegweisende Richtung könnten dabei MQAs sein, welche das Leben der Bewohner:innen nicht nur vereinfachen, sondern zudem auch im Sinne des Klimabewusstseins und der Nachhaltigkeit beeinflussen können.

Unsere Ergebnisse aus dem Projekt Stadtquartier 2050 sollen Städteplaner:innen und städtische Dienstleister:innen dabei unterstützen, neue MQAs im Quartierskontext zu entwickeln. Einerseits helfen unsere Erläuterungen, das Zusammenspiel zwischen Smart City, Bewohner:innen und MQAs genauer zu verstehen. Andererseits können unsere neun Handlungsempfehlungen auf andere Quartiersprojekte übertragen werden, um MQAs bewohnerzentriert zu entwerfen, unter ökonomischen Gesichtspunkten zu designen und unter den Gesichtspunkten des Datenschutzes regelkonform auszurollen. Trotz der Analyse der Umsetzbarkeit der Handlungsempfehlungen im Zuge der MQA im Projekt Stadtquartier 2050 innerhalb von zwei Demonstrationsquartieren bilden unsere Handlungsempfehlungen nur einen ersten, nicht abschließenden Blick auf MQAs. Gerade die technische Umsetzung von Apps als auch die Identifikation wirtschaftlicher Potenziale durch die Verwendung von MQAs könnten in weiteren Projekten näher analysiert und in Handlungsempfehlungen übersetzt werden.

In Zukunft können MQAs nicht nur ein Weg sein, Bewohner:innen mit nachhaltigen Services zu erreichen und klimabewusstes Verhalten zu fördern. Vielmehr sollten sie als ein Sprungbrett für die Entwicklung von smarten und nachhaltigen Service-Systemen oder datenbasierten Service-Plattformen verstanden werden (Häckel et al. 2021). Als Bindeglied zwischen Stadt, städtischen Dienstleister:innen und Bürger:innen hätten sie auch beispielsweise das Potenzial, das Angebot der Stadt und die Nachfrage der Bewohner:innen in neue Geschäftsmodelle zusammenzuführen und gleichzeitig einen Beitrag zur Begrenzung des Klimawandels zu leisten.
